# The impact of the parameters of the constitutive model on the distribution of strain in the femoral head

**DOI:** 10.1007/s10237-022-01678-y

**Published:** 2022-12-21

**Authors:** Sebastian Wronski, Adrian Wit, Jacek Tarasiuk, Pawel Lipinski

**Affiliations:** 1 Faculty of Physics and Applied Computer Science, AGH University of Science and Technology, 30-059, Kraków, Poland; 2 Université de Lorraine, LEM3, 7 Rue Félix Savart, 57070 Metz, France

**Keywords:** Femoral head, Constitutive model, Structure fabric tensor, Finite element method (FEM)

## Abstract

The rapid spread of the finite element method has caused that it has become, among other methods, the standard tool for pre-clinical estimates of bone properties. This paper presents an application of this method for the calculation and prediction of strain and stress fields in the femoral head. The aim of the work is to study the influence of the considered anisotropy and heterogeneity of the modeled bone on the mechanical fields during a typical gait cycle. Three material models were tested with different properties of porous bone carried out in literature: a homogeneous isotropic model, a heterogeneous isotropic model, and a heterogeneous anisotropic model. In three cases studied, the elastic properties of the bone were determined basing on the Zysset-Curnier approach. The tensor of elastic constants defining the local properties of porous bone is correlated with a local porosity and a second order fabric tensor describing the bone microstructure. In the calculations, a model of the femoral head generated from high-resolution tomographic scans was used. Experimental data were drawn from publicly available database “Osteoporotic Virtual Physiological Human Project.” To realistically reflect the load on the femoral head, main muscles were considered, and their contraction forces were determined based on inverse kinematics. For this purpose, the results from OpenSim packet were used. The simulations demonstrated that differences between the results predicted by these material models are significant. Only the anisotropic model allowed for the plausible distribution of stresses along the main trabecular groups. The outcomes also showed that the precise evaluation of the mechanical fields is critical in the context of bone tissue remodeling under mechanical stimulations.

## Introduction

As an organ, bone has a complicated structure at the macro-, meso-, and microscopic levels. For this reason, many authors have attempted to determine average material properties in line with the homogenization theory. Frequently, they applied the rules of combining two bounding models resulting from the Voigt's hypothesis of homogeneous strain (Bonfield and Li 1967) and the Reuss' hypothesis of the homogeneous stress (Piekarski 1973) fields. A mathematical model describing the microstructure of trabecular bone in two dimensions, using data from electron scanning microscopy, was first proposed by Whitehouse in 1974 (Whitehouse 1974). In that work, a polar graph representing local mechanical (elastic) properties in various directions was presented. In 1984, Harrigan and Mann expanded this approach to three dimensions, proposing the use of a second order tensor for the description of directionality in orthotropic materials (Harrigan and Mann 1984). In 1985, Cowin (Cowin 1985) developed this approach and showed that there is a theoretical relationship between the fourth order tensor of elastic constants for a porous, anisotropic material and the second order tensor describing its microarchitecture. Cowin introduced a positive-definite second order tensor, called the fabric tensor ${\varvec{H}}$, which is the stereological measure of the directional distribution of bone mass. A quantity defined in this way may be linked directly to the anisotropic properties of trabecular bone. Subsequent studies were conducted to confirm Cowin’s assumptions and develop the constructive and practical applications of this structure–property relationship (Zysset and Curnier 1995; Odgaard et al. 1997). These works showed that the eigen directions and values of fabric tensor correlate with the main directions and values of the tensor of local elastic properties of bone, expressed by the stiffness tensor ${\varvec{C}}$ or the compliance tensor ${\varvec{S}}$. They also demonstrated thus that trabecular bone may be considered as locally orthotropic medium.

Among numerous models that adopt the notion of the ${\varvec{H}}$ fabric tensor, the Zysset-Curnier model (Zysset 2003) for orthotropic trabecular bones appears to be the most universal and effective. In this approach, links are established between the results of microstructural analysis, expressed by the ${\varvec{H}}$ fabric tensor, and bone properties. Zysset and Curnier introduced the ${\varvec{M}}$ fabric tensor associated with ${\varvec{H}}$ tensor through the relation:

1 $${\varvec{M}}={{\varvec{H}}}^{-0.5}$$

Ordering the eigenvalues $${h}_{i}$$ and eigenvectors $$  \overset{\lower0.5em\hbox{$\smash{\scriptscriptstyle\rightharpoonup}$}} {h} _{i}    $$ of ***H*** in such a way that $${h}_{1}\le {h}_{2}\le {h}_{3}$$ induces the inequalities $${\mu }_{1}\ge {\mu }_{2}\ge {\mu }_{3}$$ where $${\mu }_{i}$$, are the eigenvalues of ***M*** such that $${\mu }_{i}={h}_{i}^{-0.5}$$. It appears that the eigenvector $$ \overset{\lower0.5em\hbox{$\smash{\scriptscriptstyle\rightharpoonup}$}} {\mu } _{1}  $$ coincides with direction characterized by the highest stiffness of the material. In practice, in the Zysset-Curnier model, eigenvalues of the ***M*** fabric tensor are normalized by using their mean value $$\overline{\mu }=Tr\left({\varvec{M}}\right)/3$$. The normalized eigenvalues $${m}_{i}$$ correspond to:

2 $${m}_{i}=\frac{{\mu }_{i}}{\overline{\mu }}$$

and meet the condition: *m*_1_ + *m*_2_ + *m*_3_ = 3.

The orthotropic elastic constitutive law in Zysset-Curnier model, based on the ***M*** fabric tensor, is defined by the following relations:

3 $$ E_{i} = E_{o} \rho^{k} \left( {m_{i}^{2} } \right)^{l} ,\quad \frac{{E_{i} }}{{v_{ij} }} = \frac{{E_{o} }}{{v_{0} }}\rho^{k} \left( {m_{i} m_{j} } \right)^{l} ,\quad G_{ij} = G_{o} \rho^{k} \left( {m_{i} m_{j} } \right)^{l} $$

where: $${E}_{i}$$—are the Young’s moduli along the axis (*i* = 1, 2, 3), $${v}_{ij}$$—are the Poisson’s ratios, which are defined by the ratio of strains in two perpendicular directions *j* and *i*, the direction *i* corresponding to the load (tension) direction, $${G}_{ij}$$—are the shear moduli in the direction *j* on a surface with a normal $$ \overset{\lower0.5em\hbox{$\smash{\scriptscriptstyle\rightharpoonup}$}} {e} _{i}  $$, $$\rho =BV/TV$$ – is the volume fraction of bone defined as the ratio of volume occupied by bone tissue to the volume of the entire analyzed area (ROI – Region of interest), $${E}_{0}$$, $${v}_{0}$$, $${G}_{0}$$—are equivalent to the material properties of the bone tissue (trabeculae), $$k$$, $$l$$—are the model parameters of density and anisotropy.

The anisotropy of the bone is dependent on the eigenvalues *m*_1_, *m*_2_, *m*_3_ of the ***M*** fabric tensor. It can be easily seen that in the case where *m*_*1*_ = *m*_*2*_ = *m*_*3*_ = 1, or ${M}_{ij}={\delta }_{ij}$, this model leads to an isotropic constitutive law with a value for Young’s modulus dependent on BV/TV and the Poisson’s ratio $\nu ={v}_{0}$.

To determine accurate values for the parameters of the orthotropic Zysset-Curnier model, an increasingly common approach is to compare the results of a simulation using the finite element method (FEM) with the results of mechanical tests and their mutual correlations. Expressions (3) show that the tensor of elastic constants is described by eight parameters (${E}_{0}$, ${v}_{0}$, ${G}_{0}$, *m*_1_, *m*_2_, *m*_3_, *k*, *l*) three of which (*m*_1_, *m*_2_, *m*_3_) are obtained via analysis of stereological measurements of the bone microarchitecture. The remaining five parameters are determined using a fitting procedure (regression). The local properties of the trabeculae (${E}_{0}$, ${v}_{0}$, ${G}_{0}$) are frequently evaluated using the inverse method. In this method, some initial values are assumed for the properties of the trabeculae, and subsequently, using this approximation the global properties of a particular bone microstructure (most commonly measured in microtomography) are calculated. The obtained global properties of the structure are compared with the values measured experimentally for the same bone piece. The properties of the trabeculae are modified in function of obtained differences and the global properties of the structure are recalculated. This process is repeated until measured and recalculated properties are close enough. It is assumed that the properties of the trabeculae which are consistent with this best match correspond to (${E}_{0}$, ${v}_{0}$, ${G}_{0}$) (Janc et al. 2020). Results obtained in the form of many sets of ${E}_{i}$, ${G}_{ij}$, ${v}_{ij}$,$\rho$, ${m}_{i}$, are used as input data for the optimization problem to determine the parameters *k* and* l* from the experimental data obtained for bones with various BV/TV values.

The moduli determined by the model allow for the generation of the full form of the stiffness tensor ***C*** or the compliance tensor ***S*** which considers the symmetry of orthotropic materials. The formula (3) provides a form for the compliance matrix for this type of material:

4 $$\left[{\varvec{S}}\right]=\left[\begin{array}{cccccc}\frac{1}{{E}_{1}}& -\frac{{\nu }_{21}}{{E}_{2}}& -\frac{{\nu }_{31}}{{E}_{3}}& 0& 0& 0\\ -\frac{{\nu }_{12}}{{E}_{1}}& \frac{1}{{E}_{2}}& -\frac{{\nu }_{32}}{{E}_{3}}& 0& 0& 0\\ -\frac{{\nu }_{13}}{{E}_{1}}& -\frac{{\nu }_{23}}{{E}_{2}}& \frac{1}{{E}_{3}}& 0& 0& 0\\ 0& 0& 0& \frac{1}{{G}_{23}}& 0& 0\\ 0& 0& 0& 0& \frac{1}{{G}_{31}}& 0\\ 0& 0& 0& 0& 0& \frac{1}{{G}_{12}}\end{array}\right]$$

In many works concerning determination of the states of stress and strain occurring in bones, only a selected fragment of a bone or limb is considered due to simplification. The load is expressed in the form of forces concentrated on and applied to nodes of a finite elements mesh. Such an approach has been applied, among others, in works of (Chen et al. 2015) or (Marco et al. 2019). The obtained results would appear to be very sensitive to boundary conditions and/or the loads applied. Minor changes in the directions of the forces acting upon the structure, or in the selection of nodes where these forces are applied, could cause significant changes in the local distribution of strains and stresses. Many works in this subject area (particularly when it comes to simulations of loads on the femur) make use of the Orthoload (https://orthoload.com/.) database, which specifies forces supported by the femoral head during typical activities such as walking or climbing. Such an approach has been used for instance in works (Yosibash et al. 2015) and (Hazrati Marangalou et al. 2015). In these papers, the load supported by the entire femoral head was reduced to a single resultant force. The femoral head is the site of attachment for many muscles and tendons, so in order to obtain a reliable estimate of the state of mechanical fields, these points of attachment and directions of muscular actions must be precisely described. Calculations which take into account such a distribution of forces have been presented, among other, in (Latifi et al. 2014). A model so defined provides a reliable image of the internal state of the bone, similar to its actual behavior. However, it is worth noting that in the works mentioned above, the applied loads were stationary. Attempts have been made to take into account non-stationary and even dynamic loads, as in the papers (Carter et al. 1987) and (Hambli et al. 2013), although these are still rough approximations of time dependent muscular activities during walking.

The definition of the bone elastic properties is a second crucial decision. In this paper, a Mean Intercept Length (MIL) concept is used for the description of heterogeneous and anisotropic bone properties. Based on MIL results, ellipsoids were generated describing the anisotropy of the microstructure associated with each finite element. The ***M*** fabric tensors was deduced from each ellipsoid and its eigen values and directions computed.

A finite element model (mesh) was constructed in which the forces applied were chosen so that their components in the bone coordinate system reflected the complex muscular load to which this organ is subjected. In nature, the muscle is attached to the bone by a tendon in such a way that its action is spread over a certain area of the bone. The points at which force is applied in a numerical model correspond to the anatomical sites of muscle attachment, allowing for the proper modeling of internal states arising during selected phases of the normal walking cycle. To prevent numerical artifacts resulting in excessive concentration of stresses, additional layers of elements were created which aim to play the role of tendons and cartilage at the anatomical sites of muscular attachments and contact with other organs. Preprocessing, processing and post-processing tasks were conducted in the Simulia environment (Hibbitt, Karlsson & Sorensen, Inc., 2000) using Abaqus ver. 6.13 software. Three material models were tested with different properties of porous bone carried out in literature: a homogeneous isotropic model, a heterogeneous isotropic model, and a heterogeneous anisotropic model. In three cases studied, the elastic properties of the bone were determined basing on the Zysset-Curnier approach.

The principal aim of the work was to study the influence of the considered anisotropy and heterogeneity of the femoral head on the mechanical fields, such as stresses, strains, or elastic strain energy density, during a typical gait cycle. The precise description of these quantities is crucial in modeling of bone remodeling process.

## Materials and methods

### Finite element models

**Fig. 1:**
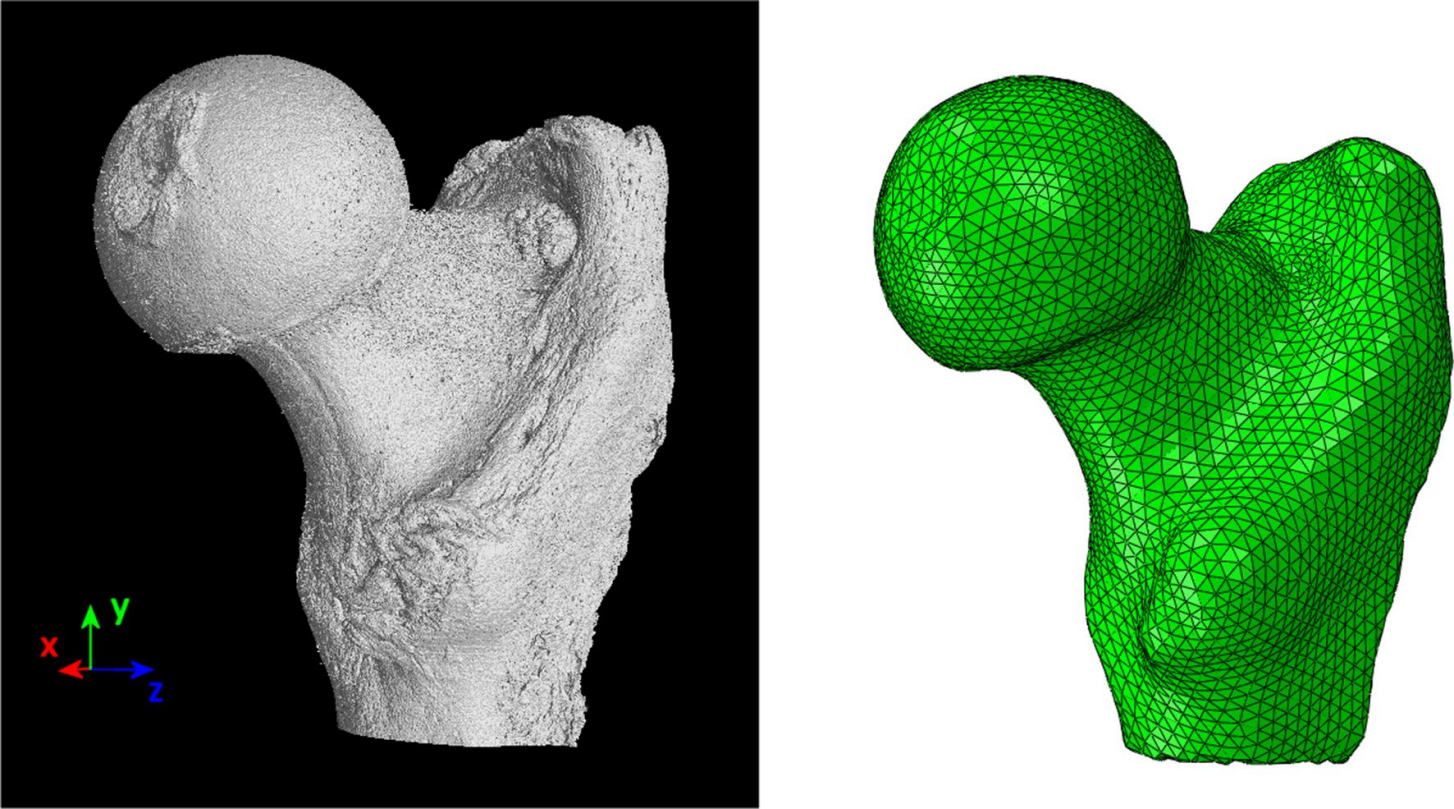
Three-dimensional presentation of tomographic data for the femoral head and mesh of elements

The FEM model of the femoral head was drawn from the database mentioned earlier (Virtual Physiological Human Project). This database contains 33 measurements of femoral heads of donors whose average age was 77.8 ± 10.0 years. The measurements of all bones were made using a Scanco Medical tomograph (Scanco Medical AG, Brüttisellen, Switzerland). For the analysis presented in this paper, the bone labeled FEM01567 (Javad Hazrati-Marangalou 2013) was used, generated from micro-CT scans with an isotropic resolution of 82 µm. The bone was made available in the form of binarized images. This same database provided access to post-binarization tomographic measurements, and a finite element mesh made up of 97 384 tetrahedral elements of the C3D4 type in the Abaqus convention. The geometry of the bone (tomographic data) and its finite elements mesh are shown in Fig. 1. The mean element size was about 2.5 mm, to be consistent with the resolution of the tomograph used and the window size for the MIL calculation. This size was fixed thanks to the local analysis of bone parameters such as porosity, size of trabeculae, and average distances between them. It enables reproducing correctly the natural spatial variability of the trabecular microstructure of the bone.

**Fig. 2:**
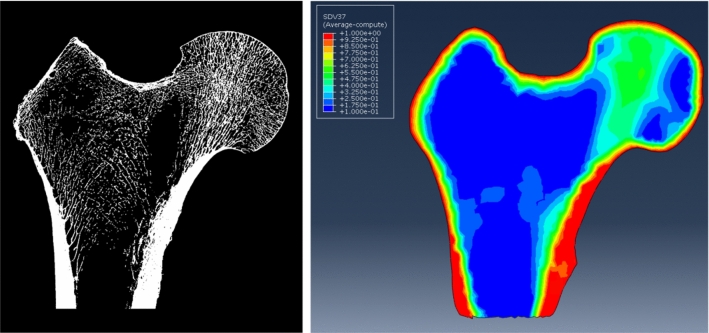
Sample intersect-section of the femoral head and BV/TV parameter distribution assigned to a finite element mesh

To each element of the mesh were assigned parameters such as volume fraction (BV/TV) and quantity describing the internal structure of the bone (***H*** fabric tensor) which are essential for the correct definition of its material properties in accordance with the Zysset-Curnier model. A sample binarized cross section of the femoral head and volume fractions assigned to the relevant elements of the mesh are shown in Fig. 2.

The parameters of the model necessary for the calculation of the ***S*** tensor, i.e., ${E}_{0}$, ${v}_{0}$, and ${G}_{0}$ as well as $k$ and $l$, were taken from the literature (Gross et al. 2013) and are summarized in Table 1.

**Table 1:** Material constants used in calculations

${{\varvec{E}}}_{0}$[MPa]	${{\varvec{v}}}_{0}$[−]	${{\varvec{G}}}_{0}$[MPa]	K [−]	L [−]
22,500	0.3	8650.0	1.9	0.99

Similar values were used in other works devoted to the description of properties of trabecular structures, see for instance (Chevalier et al. 2009) or (Charlebois et al. 2010).

Using Eq. (3), a compliance matrix was determined for each element of the mesh. Because of the tomograph resolution, for high BV/TV values, typical for compact cortical bone, the trabecular structure disappears and the values of the three MIL eigenvalues approach 1. The constitutive relation deduced from Zysset-Curnier model becomes isotropic. In the present work, it was assumed that for areas with the BV/TV fraction exceeding 0.9, the properties are isotropic and correspond to that of the compact cortical bone of BV/TV = 0.95. In turn, for very low BV/TV values, the trabecular structure becomes so porous that the determined MIL values do not describe the anisotropy of the structure, but simply the orientation of individual trabeculae. Also, for this reason, in areas of bone with BV/TV values lower than 0.1, the isotropic properties of the material were assumed corresponding to a constant bone density reflected by the value of BV/TV.

The constitutive model of bone was implemented using User MATerial (UMAT) subroutine. Before calling the appropriate UMAT subroutine, a Solution-Dependent Variables INItialization (SDVINI) routine was executed aiming to assign values to materials constants. In this subroutine, data are read for each element, including the volume fraction and eigenvectors of the ***M*** tensor. Basing on Zysset-Curnier model, the **S** tensor was determined for each element. The components of the stiffness matrix ***C***, obtained by inversion of ***S***, were recorded in the STATE Variables (STATEV) and sent to the first step of FE computation.

**Fig. 3:**
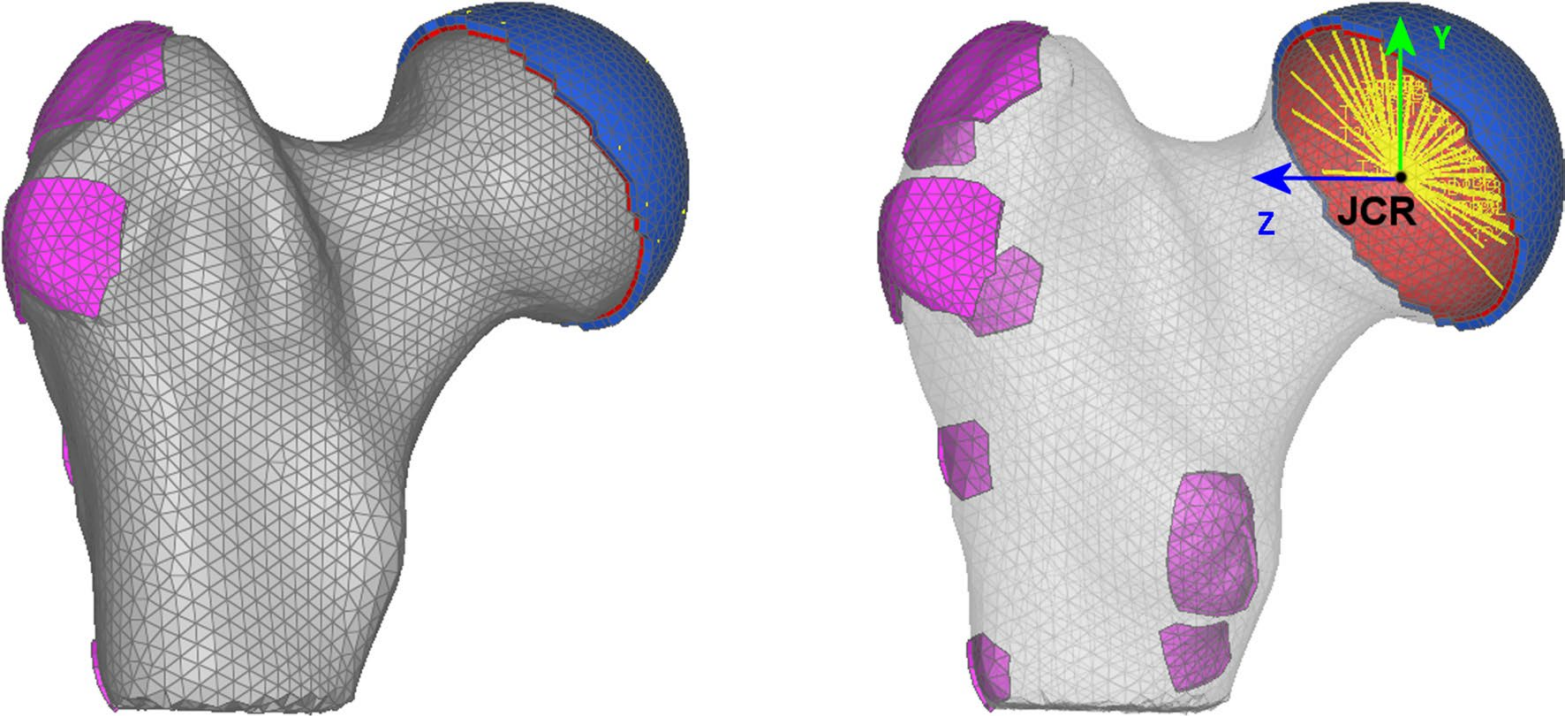
Distribution of elements which play the role of tendons and cartilage, and load on the hip joint. An additional layer of elements at sites of muscle attachment allows local concentrations of strains to be avoided

Point forces applied directly to the nodes of the bone mesh may lead to an unrealistic stress concentration at the site of their attachments. To smooth out the local influence of forces, additional layers of elements were generated to play the role of tendons and cartilage. Figure 3 presents the distribution of elements symbolizing the role of tendons and cartilage (in pink) and the layer of stiff elements (in blue) simulating the cotyloid cavity.

The mechanical properties of the elements representing the tendons and cartilage were supposed elastic and isotropic. Their Young’s modulus and Poisson’s ratio were taken from the literature, (Kot et al. 2012) and (Chen et al. 1996). In the calculations, the following values were assumed for tendons and joint cartilage: E = 5 MPa, ${v}$=0.3.

The actions of individual muscles were evaluated from inverse kinematics calculations performed in OpenSim using the model Gait2392 created by Thelen (Delp et al. 2007). The value of the force of each muscle attached to the femur was extracted. For this purpose, the “MuscleForceDirection” plug-in (Phillips et al. 2015) was used to obtain access to the topography of the attachment points of objects representing muscles and their direction of actions (force of muscle contraction). These values were then assigned to the FEM model. The name of muscles and values of muscular forces applied to the model for two leg configurations corresponding, respectively, to 30% and 60% of the gait cycle are summarized in Table 2.

**Table 2:** Muscular forces (N) applied to the model for two leg configurations corresponding, respectively, to 30% and 60% of the gait cycle

Muscle name (according to OPENSIM nomenclature)	Configuration 1				Configuration 2			
	Direction			Magnitude (*N*)	Direction			Magnitude (*N*)
	*n*_*x*_	*n*_*y*_	*n*_*z*_		*n*_*x*_	*n*_*y*_	*n*_*z*_	
Glut_max1	− 0.481	0.851	− 0.208	21.547	− 0.082	0.145	0.170	3.450
Glut_max2	0.473	0.852	0.221	4.851	0.473	0.852	0.221	5.516
Glut_max3	− 0.454	0.719	− 0.525	3.017	− 0.454	0.719	−0.525	4.722
Glut_med1	0.398	0.885	− 0.238	240.442	0.336	0.928	− 0.155	7.113
Glut_med2	0.050	0.858	− 0.510	135.955	− 0.049	0.899	− 0.433	6.153
Glut_med3	− 0.205	0.701	− 0.682	119.839	− 0.309	0.705	− 0.637	163.206
Glut_min1	0.333	0.802	− 0.495	36.459	0.277	0.851	− 0.445	2.540
Glut_min2	0.173	0.810	− 0.559	39.606	0.106	0.851	− 0.512	2.767
Glut_min3	− 0.011	0.746	− 0.664	37.537	− 0.932	0.774	− 0.626	10.013
Iliacus	0.892	0.331	− 0.305	114.663	0.892	0.331	− 0.305	228.926
Pect	0.346	0.719	− 0.601	2.423	0.323	0.747	− 0.580	10.798
Perif	− 0.424	0.466	− 0.776	24.329	− 0.504	0.431	− 0.748	38.924
Psoas	0.877	0.387	− 0.283	130.575	0.877	0.387	− 0.283	261.665
Quadfem	0.002	− 0.182	− 0.983	3.729	0.076	− 0.181	− 0.980	111.287
Gem	− 0.401	− 0.231	− 0.886	1.778	− 0.360	− 0.282	− 0.888	15.389

It should be emphasized that the force directions are defined in femur bone coordinate system defined in OpenSim. In this coordinate system the coronal plane of the bone head is parallel to the YZ plane. The origin of the coordinate system is at the center of the bone head (see Fig. 1 and 3). The reaction force components in the hip joint center were distributed to the stiff surface elements representing the cotyloid cavity by using rod elements as represented in Fig. 3b.

This aimed to distribute the load onto the surface of the femoral head in contact with the concave surface of the cotyloid cavity. A similar approach was also used in the paper of (Phillips et al. 2015). The forces of contact of the joints were calculated at the center of the joint using the “Joint Reaction” plug-in available in OpenSim (Steele et al. 2012). The use of such a simplified method ensures a significant shortening of computation time in comparison with the consideration of contact over the surface of the joints. The reaction force corresponding to two leg configurations studied are resumed in Table 3.

**Table 3:** Reaction force components for both leg configurations

Reaction force components	Configuration 1				Configuration 2			
	Direction			Magnitude (*N*)	Direction			Magnitude (*N*)
	*n*_*x*_	*n*_*y*_	*n*_*z*_		*n*_*x*_	*n*_*y*_	*n*_*z*_	
Joint Reaction Loads	− 0.153	− 0.942	0.298	1383.005	− 0.288	− 0.789	0.541	773.691

**Fig. 4:**
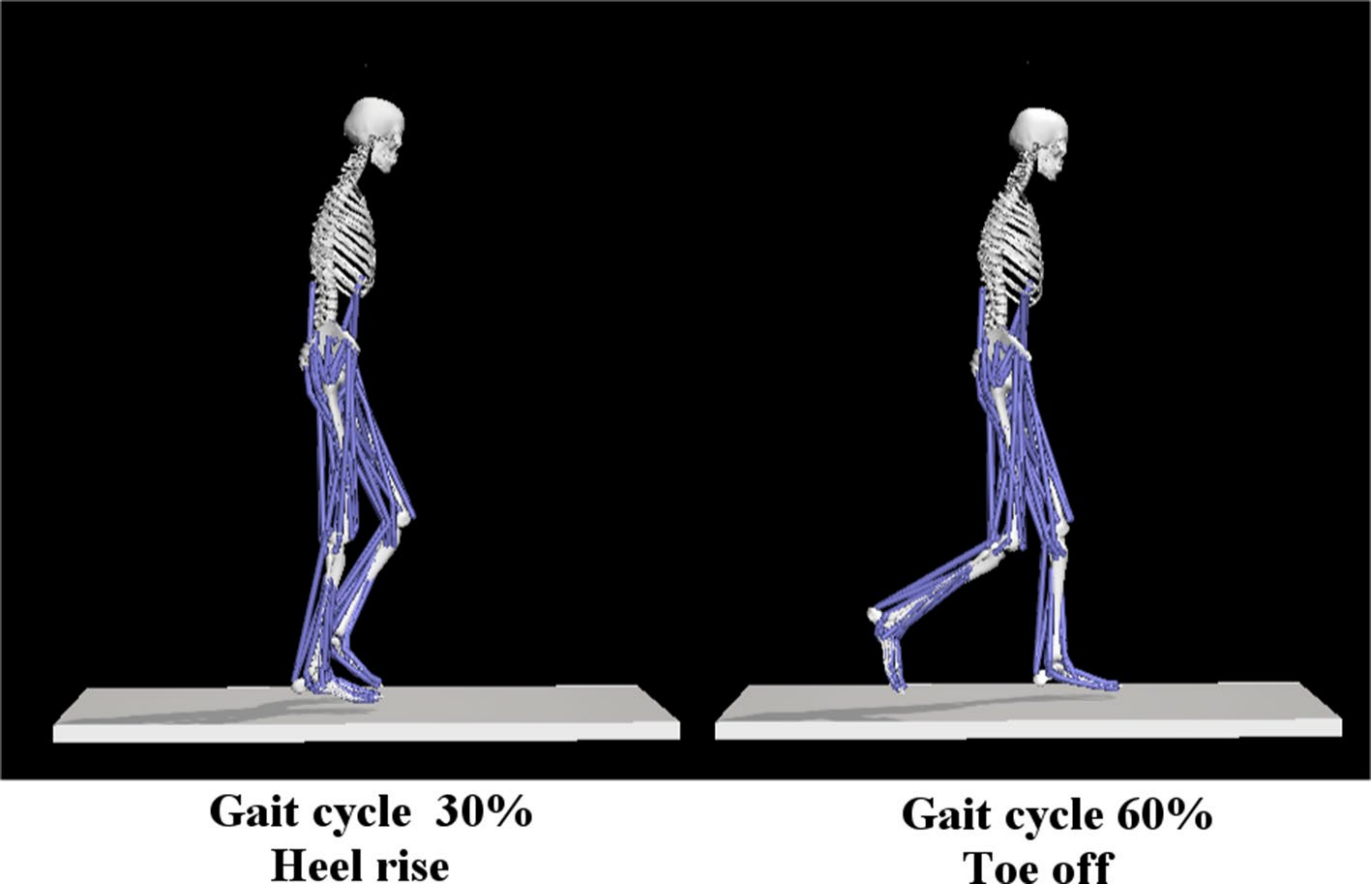
Gait phases analyzed for distribution of stresses in the femoral head. Two instants of movement were selected for analysis: Heel rise—the moment in which begins the motion of the leg backwards. Toe-off moment in which the leg is raised toward the back and the femur is parallel to the vertical axis, while the leg is not loaded

In this paper, the right femur was analyzed at two moments of motion, namely in a phase during which there occurs substantial load on the limb (during contact with the floor, the entire weight of the body is borne by the limb) and a phase during which the limb does not have contact with the floor. In both cases, the right femur is parallel to the axis of the body; thus, the orientation of the principal stresses should be like one another, facilitating comparison of their values at both instants of motion. The analyzed phases of motion during the gait cycle are presented in Fig. 4.

### Methods

The impact of the type of the constitutive model on the predicted distribution of stresses and strains in the femoral head, for two selected moments of gait, was studied in this paper. Three material models were constructed with different properties of the porous tissue:

Model 1—heterogeneous and anisotropic: mechanical properties are orthotropic and determined in accordance with Zysset’s model. Their anisotropy results from the orientation of the principal directions and values of the ***M*** and the volume fraction BV/TV.

Model 2—heterogeneous and isotropic: the density of porous tissue in all elements was estimated through BV/TV volume fraction determined from a binarized tomographic images of the bone. As a result, the values of Young’s modulus in various regions are different and depend on the estimated porosity. This value varies from 280 MPa for bone having BV/TV $\le$ 0.1 to 18420 MPa for bone with a BV/TV $\ge$ 0.9. As the bone is isotropic, its Poisson’s ratio is $\nu ={\nu }_{O}=0.3$ in accordance with Zysset model.

Model 3—homogeneous and isotropic: in this model, a constant porous bone density was assumed as resulting in homogeneous and isotropic mechanical properties for this tissue. The value of Young’s modulus was determined according to expression (2), using for this purpose the mean BV/TV volume value of the analyzed area of the bone. This was $\overline{BV/TV}=0.27$ and its corresponding value for Young’s modulus was equal to $\overline{E}=1835\mathrm{MPa}$. As above, since the properties of this bone are, by hypothesis, isotropic:$\nu ={\nu }_{O}=0.3$.

In these three material models, matching to the assumptions of Zysset, isotropic elastic properties with a constant value of Young’s modulus were assumed for cortical bone and Poisson’s ratio associated corresponded to the value of $BV/TV=0.95$. They were, respectively:$E=22500MPa$ and $\nu ={\nu }_{O}=0.3$.

**Fig. 5:**
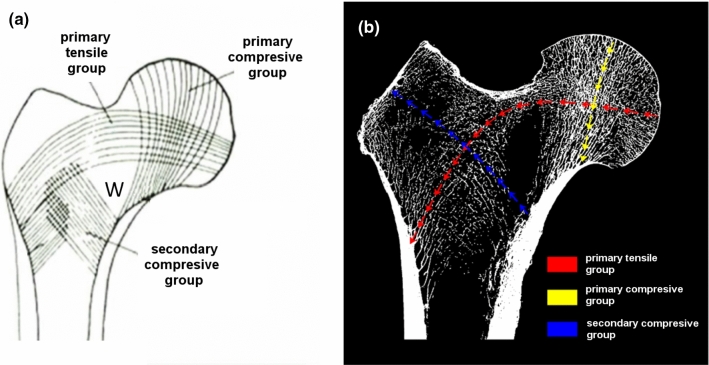
Trabecular patterns in the proximal femur. **a** Lines in diagram show the main direction of trabecular groups and Ward’s triangle inside. **b** Right hip radiogram with defined colored paths along which the computation results are analyzed

In the femoral head, a set of areas can be distinguished with diverse spatial distribution of trabeculae as illustrated by Fig. 5a (Shivji et al. 2015). This results from the fact that the distinctive microstructure of the bone is optimized in terms of stresses and strains due to many daily activities. The femoral head is practically free of torsional stresses thanks to a spherical type of connection with the pelvis. The neck of the femur is submitted to the elevated compressive stresses. This is reflected in its locally dense mineralization. In the study (Basharat et al. 2015), it was pointed out that the absolute value of compressive stress acting on the neck of the femur is twice as great as the tensile stress. The greater trochanter is composed of thin and barely visible trabeculae working mainly in extension. The central area, marked with the letter *W* in Fig. 5a, with a characteristic arrangement of struts, is called Ward’s triangle. This area of femoral neck is very porous.

The above microarchitecture is reflected by the occurrence of specific, easily identified bands in which the bone trabeculae are arranged in such a way as to best bear external loads. In the literature, four bands are often differentiated in the femoral head. The two of them experience intensive compression load, and the two remaining undergo tensile stresses. They are well schematized in Fig. 5a.

The first band of the *primary compressive group* stretches from the medial cortex of the neck of the femur to the upper part of the femoral head. The *primary tensile group* extends from the lateral cortex and ends in the lower part of the femoral head. The third band known as the *secondary compressive group* stretches upwards and sideways, from the lower part of the femur below Adams arch in the direction of the greater trochanter and the upper neck of the femur. The band known as the *secondary tensile group* combines the areas of the lateral cortex with the area of the neck of the femur. This group bears the load of the hip joint through the femoral head in the direction of the medial cortex.

In this study, the distributions of stresses and strains along three paths shown in Fig. 5b) were studied, namely the paths passing through the center of the *primary compressive group* (path 1), *primary tensile group* (path 2) and *secondary compressive group* (path 3). Path (3) was artificially lengthened to the surface of the bone (the surface of the greater trochanter).

The correct prediction of the fields of stresses and strains is especially important in the context of the metabolism of bone tissue. The bone tissue can change its microarchitecture (but also external shape) in function of mechanical loads it bears. In the course of such a process, there is a remodeling of the bone tissue resulting from subsequent actions of osteoclasts and osteoclasts (Sikavitsas et al. 2001). In accordance with Frost’s model (Frost 2003), four thresholds of mechanical stimulus should be introduced for the description of mechanostat principles, namely MESr, MESm, MESp, and Fx, where the abbreviation MES stands for Minimally Effective Strains. These values determine zones of bone formation, or modeling, (values above MESm) and of its resorption (values below MESr). If the stimulus is within the zone delimited by MESr and MESm, the tissue is in balance and does not change its density. This area is known as the dead or lazy zone. The principal data of Frost model are summarized in Table 4 in the form of threshold values for strains, stresses, and density of elastic strain energy.

**Table 4:** Suggested values for MES (Frost 2003)

		Strain value [$\mu$ strain]	Stress value [MPa]	Strain Energy density [MPa]
MESr	The threshold range for disuse mode bone remodeling	50–100	1–2	2.8 10^−5^–11.25 10^−5^
MESm	Threshold for bone remodeling	1000–1500	20	0.01125–0.02531
MESp	Bones operational microdamage threshold range	3000	60	0.10125
Fx	Bones ultimate strength and fracture strength	25,000	120	$\sim$ 1.5

The determination of local strains and stresses requires knowledge of the localization tensors of these tensorial quantities. Their theoretical determination is not simple and requires in-depth knowledge of the microarchitecture of the bone (Janc et al. 2020).This problem can be side-stepped by analyzing scalar quantities. Many models of adaptation and remodeling of bone use the elastic strain energy density *w* as a stimulus. The dependency between the local *w* and global *W* values of this energy is simple:

5 $$W={f}_{t}w\approx \mathrm{BV}/\mathrm{TV }w$$

where ${f}_{t}$ is the volume fraction of bone tissue. This expression is valid in the case of a bi-phasic materials in which one of the phases is empty (as in the case of bone). Therefore, it is possible to determine the local strain energy density knowing its mean value determined by the FE method. We thus have:

6 $$w={\left(\mathrm{BV}/\mathrm{TV}\right)}^{-1}\mathrm{W}$$

The threshold values of elastic strain energy density $w$, which in many studies is assumed as a mechanical stimulus, was determined from strain thresholds using Young’s modulus of compact bone tissue (*E* = 22,500 MPa). They are summarized in the last column of Table 2.

Numerous data from the literature indicate that for stresses in the range of 2–20 MPa, the tissue is in a state of balance. Likewise, outside of this range remodeling of bone tissue takes place. These values suggest that error of just a few MPa in computed stresses can alter the results of adaptation process simulation. It is worth noting that the authors do not specify whether the ranges provided correspond to components, principal or effective stresses (such as von Mises equivalent stress). In function of the stimulus changes, the bone modifies its density accompanied by a reorientation in the space of its trabeculae (Geraldes et al. 2016). This process can be interpreted at global bone level as an alteration of bone mechanical properties described by the evolution of the stiffness tensor and a reorientation of the axes of material symmetry. For this reason, also, the correct determination of the principal directions of strain or stress tensor seems to be crucial when modeling the bone adaptation process.

## Results

In this section, the main results concerning stress and strain fields as well as the strain energy density field in femoral head are presented for two configurations of the leg illustrated by Fig. 4.

### Stress and strain states versus material models of femoral porous bone

**Fig. 6:**
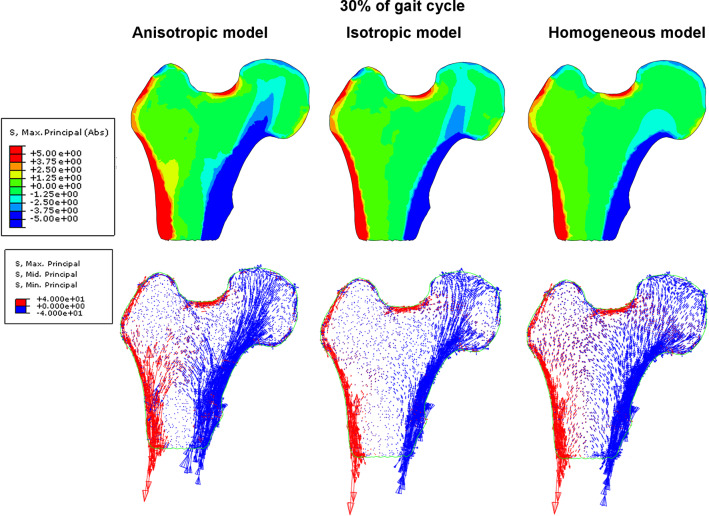
Distribution of major principal stress and eigen vectors of the stress tensor inside the femoral head for three models of elasticity at 30% of the gait cycle

**Fig. 7:**
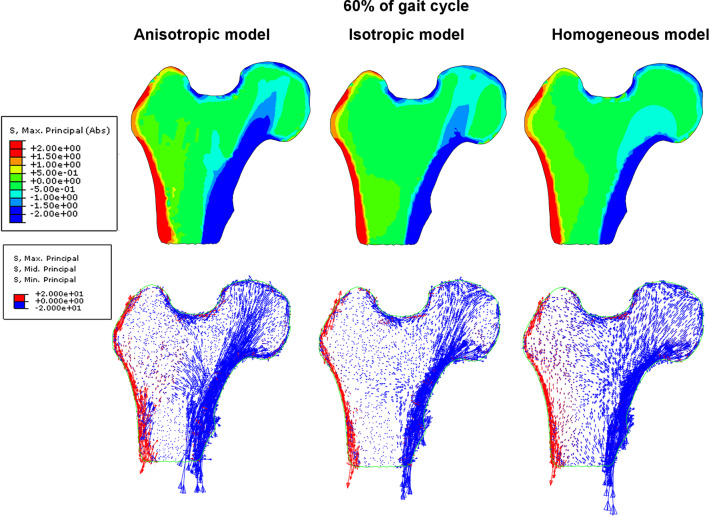
Distribution of major principal stress and eigen vectors of the stress tensor inside the femoral head for three models of elasticity at 60% of the gait cycle

Figures 6 and 7 illustrate, for the three constitutive models of trabecular bone, the field of principal stresses in the coronal (frontal) plane passing through the middle of the femoral head. The results drawn in Fig. 6 concern the instant of heel rise (30% of gait cycle). First column illustrates stresses obtained with heterogeneous and anisotropic Hooke's law of porous bone, the second one with the heterogenous but isotropic constitutive relation for this tissue and the third one those for homogeneous and isotropic porous bone.

The images in the first row show the color maps of the major principal stress. The major principal stress (Max. Principal (abs) in Abaqus) is the largest absolute value among three principal stresses. The second row show the distributions of vectors of principal stresses. The legend on the corresponding images has been chosen so that compressive stresses (negative), regardless of their values, are drawn in blue, while tensile stresses (positive) are drawn in red. The length of vectors indicates their intensity.

At 30% of the gait cycle, significant differences between the models occur in the shaft of the femur as well as in the femoral head. The anisotropic model predicts broad bands of heavily loaded cortical bone and adjacent porous tissue. The width of these bands of bone decreases by almost twice when comparing the anisotropic and homogeneous models. In the case of the isotropic heterogeneous model, the cortical bone locally bears greater stresses than in the anisotropic model. For the Zysset anisotropic model the distribution of the stress in the trabecular bone is highly contrasted (nonuniform) and reflects the spatial microstructure of the trabeculae arrangements. The heterogeneous isotropic model mirrors only partially the internal structure of the trabeculae. In this case, the *primary compressive group* is quite visible, but the level of compressive stresses occurring in it is much lower than in the case of the anisotropic model. The homogeneous isotropic model of trabecular bone “smooths” the stresses in the entire volume of the porous bone. The vector fields of the principal stresses make clear this observation. In the anisotropic model, the compressive load is carried by the cortical bone and characteristic arches in the trabecular tissue which are formed by the bone struts illustrated in Fig. 5. For the 60% of the gait cycle (toe-off moment), the differences between models are equally significant as illustrated by Fig. 7. The external load carried by the bone is lower at this leg configuration, yet the anisotropic model allows for the identification of the existence of characteristic trabecular groups with an anatomically parallel arrangement of struts of femoral head. The heterogeneous isotropic model also reflects for this configuration the *primary compressive group*, although the stresses occurring within it are significantly lower than in the anisotropic model. The situation is notably different in the case of the homogeneous isotropic model, in which the stresses in the shaft of cortical bone remain relatively high, yet the spongy matter also takes part in bearing the bending moment due to the load on the femoral head. In this last case the stress distribution does not reflect the arches of trabeculae. Rather, the entire area of trabecular bone can be divided into two domains: a compressive one on the lateral side of the bone and a tensile zone on the opposite medial side.

Another area with high divergence in stresses predicted by the models is the region where the shaft of the bone meets the femoral epiphysis. In this area, the *secondary compressive group* and *secondary tensile group* begin. Results have shown that the anisotropic model predicts substantial stresses in this area which do not occur in the predictions of the other two models. In the case of 60% gait cycle configuration, the differences among the models are not so significant. The stresses are, as a rule, twice lower than for the phase of full load.

**Fig. 8:**
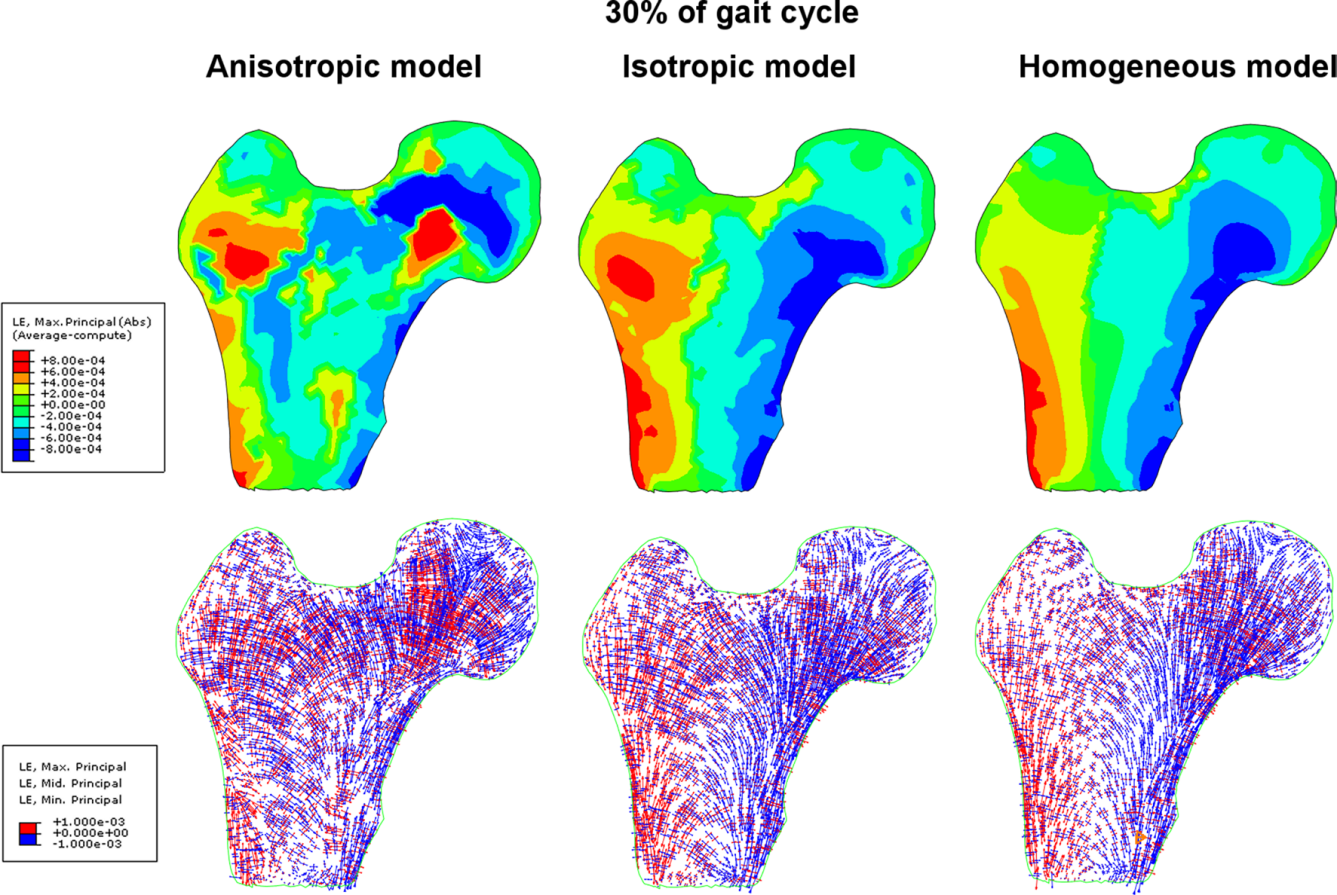
Max Principal strain (Abs) fields and main vectors of strain tensor inside the femoral head for three material models of trabecular bone at 30% of the gait cycle

**Fig. 9:**
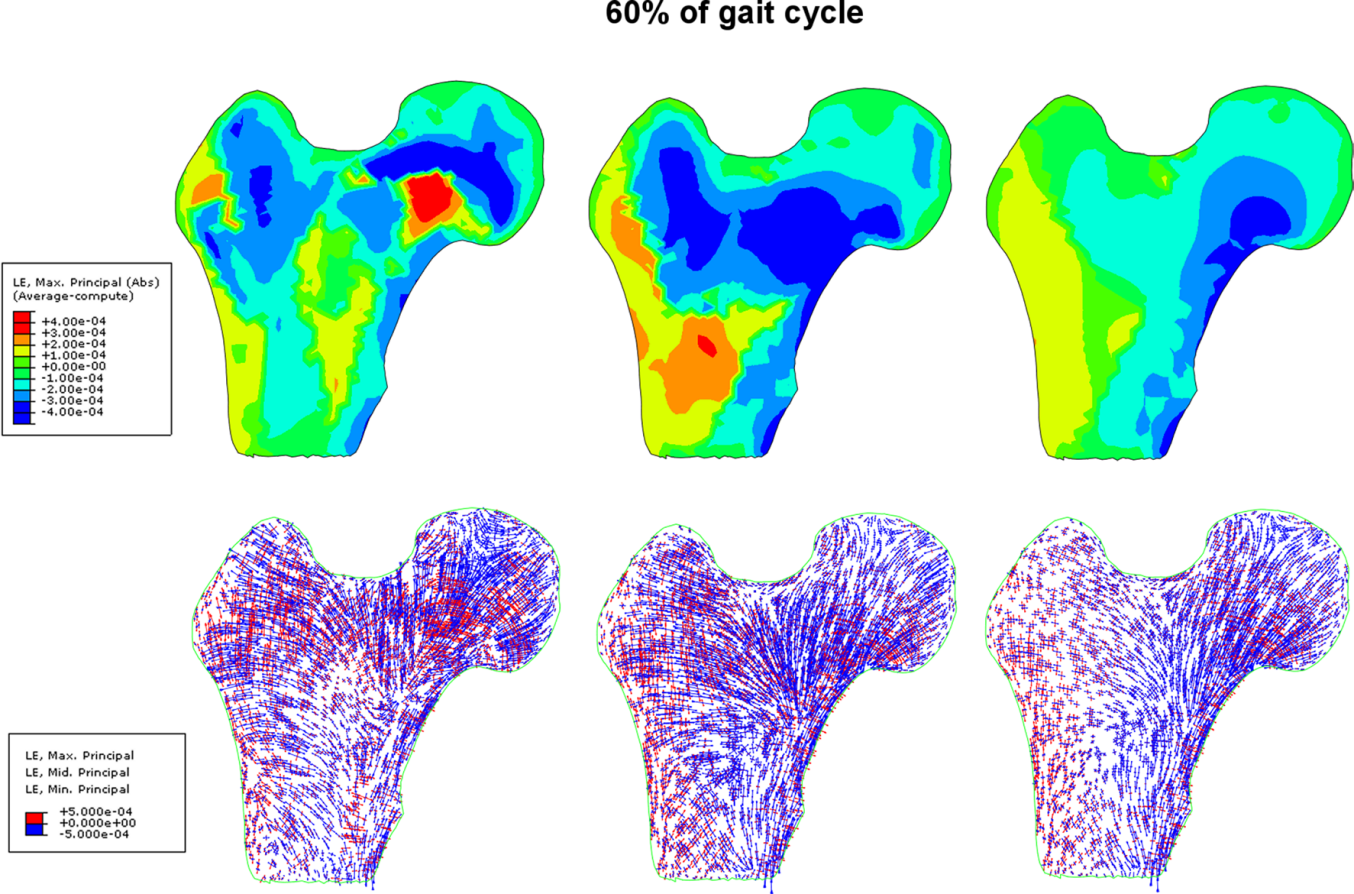
Max Principal strain (Abs) fields and main vectors of strain tensor inside the femoral head for three material models of trabecular bone at 60% of the gait cycle

A second very important mechanical internal variable, often used in models of bone tissue remodeling, is the strain tensor. Frost’s original approach is based on this quantity. Below, thus, are presented color maps of major principal values and vectors of strain tensor. The major principal strain is defined in the same manner as the major principal stress. The results are illustrated by Figs. 8 and 9, respectively, at 30% and 60% of the gait cycle. These figures are organized similarly to Figs. 6 and 7 illustrating the principal stress fields. As a result of three simulations, for first leg configuration (Fig. 8), the major principal strain fluctuates within a range of −0.00095 to 0.0008. The anisotropic model predicts slightly more intense deformation of trabecular bone, in particular near the center of the femoral head. The homogenous and heterogeneous isotropic models predict high strains mainly on the lateral and medial sides of the femur shaft. The fibers of the lateral side are elongated whereas those on the medial side are shortened.

These strains are at least twice less intense for the second leg configuration illustrated in Fig. 9. The major principal strain varies between −0.0004 and + 0.0004. In the case of the anisotropic and homogeneous models, the color maps of this strain are similar for both configurations studied. The fields predicted by the heterogeneous isotropic model are considerably different. When the leg is in contact with the ground, the greatest strains occur in cortical bone. In the second limb configuration, the trabecular bone inside the femoral head and shaft are strained more intensely than the cortical bone.

The distribution of eigenvectors of strain tensor also differs considerably from model to model but their fields (not intensity) are similar for both leg configurations. In the anisotropic model, significant positive and negative strains take place inside the femoral head. The vicinity of primary compressive group trabeculae is especially interesting. The compressed trabeculae of this group (blue vectors) are surrounded by the trabeculae undergoing elongation (red vectors). The homogeneous model leads to a clear differentiation of the lateral and medial sides. On the medial side the bone is subjected to intense negative strain in the direction tangential to the shaft. On the lateral side the strains are positive and tangent to the cortical bone. The heterogeneous isotropic model predicts negative strains in the neck of the femur. The corresponding vectors are parallel to primary compressive group of trabeculae. By comparing the fields of eigenvectors of the stress and strain tensors (Figs. 6 with 8 and 7 with 9), it clearly appears that maximal stresses (in absolute) are mainly concentrated in the regions belonging to the three groups of trabeculae. Comparing to the stress fields, the strain fields seem to be more "disordered" for three material models analyzed and especially for the model based on the Zysset's constitutive law. Also, Figs. 6, 7, 8, and 9 testify that the eigenvectors of stress and strain tensors generally do not coincide. It means, the direction of maximal stretching (shortening) of the bone is different from the direction of the maximal tension (compression).

### Strain energy density versus material models of femoral porous bone

**Fig. 10:**
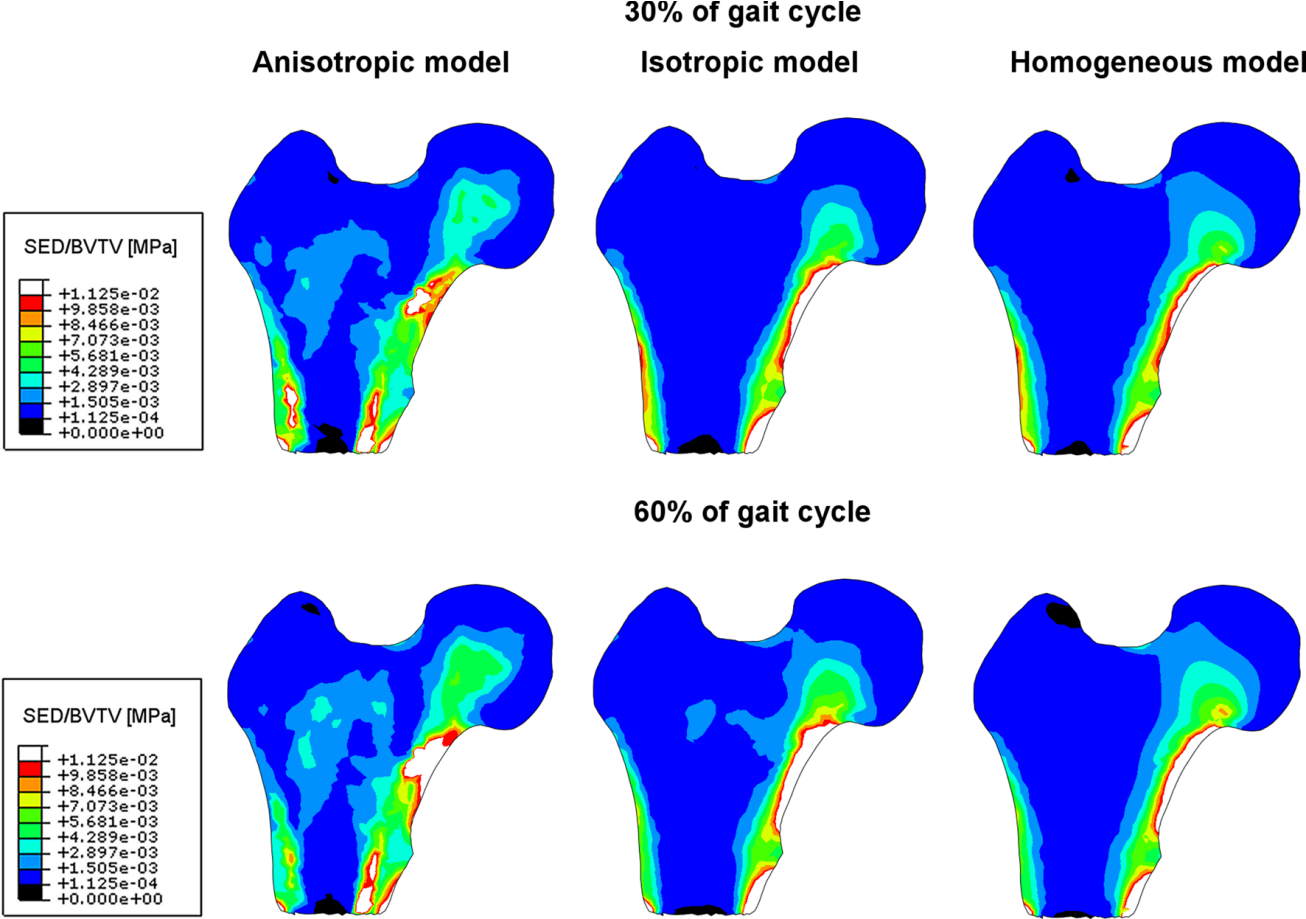
Color maps of elastic strain energy density inside the femoral head for three constitutive models and two limb configurations (30% and 60% of the gait cycle)

Figure 10 presents the fields of the strain energy density defined by expression (6) for both configurations of leg. The lower and upper limits of the legend of this figure correspond, respectively, to MESr = 1.125 10^–4^ MPa and MESm = 1.125 10^–2^ MPa, values which are specified in Table 2.

This figure proves that for the three models and both leg configurations, the $w$ sits in the Frost’s dead zone apart from small white areas, for which $w\ge \mathrm{MESm}$, located mainly in cortical bone, and black zones, for which $w\le \mathrm{MESr}$, situated mainly in the central part of the bone shaft. It is worth noting that for 30% of the gait cycle in the case of the anisotropic material model, the field of energy density, like the field of major strain of Figs. 8 and 9, is more uniform than in the case of the isotropic models.

## Discussion

### Anisotropy and inhomogeneity of the femoral hear

**Fig. 11:**
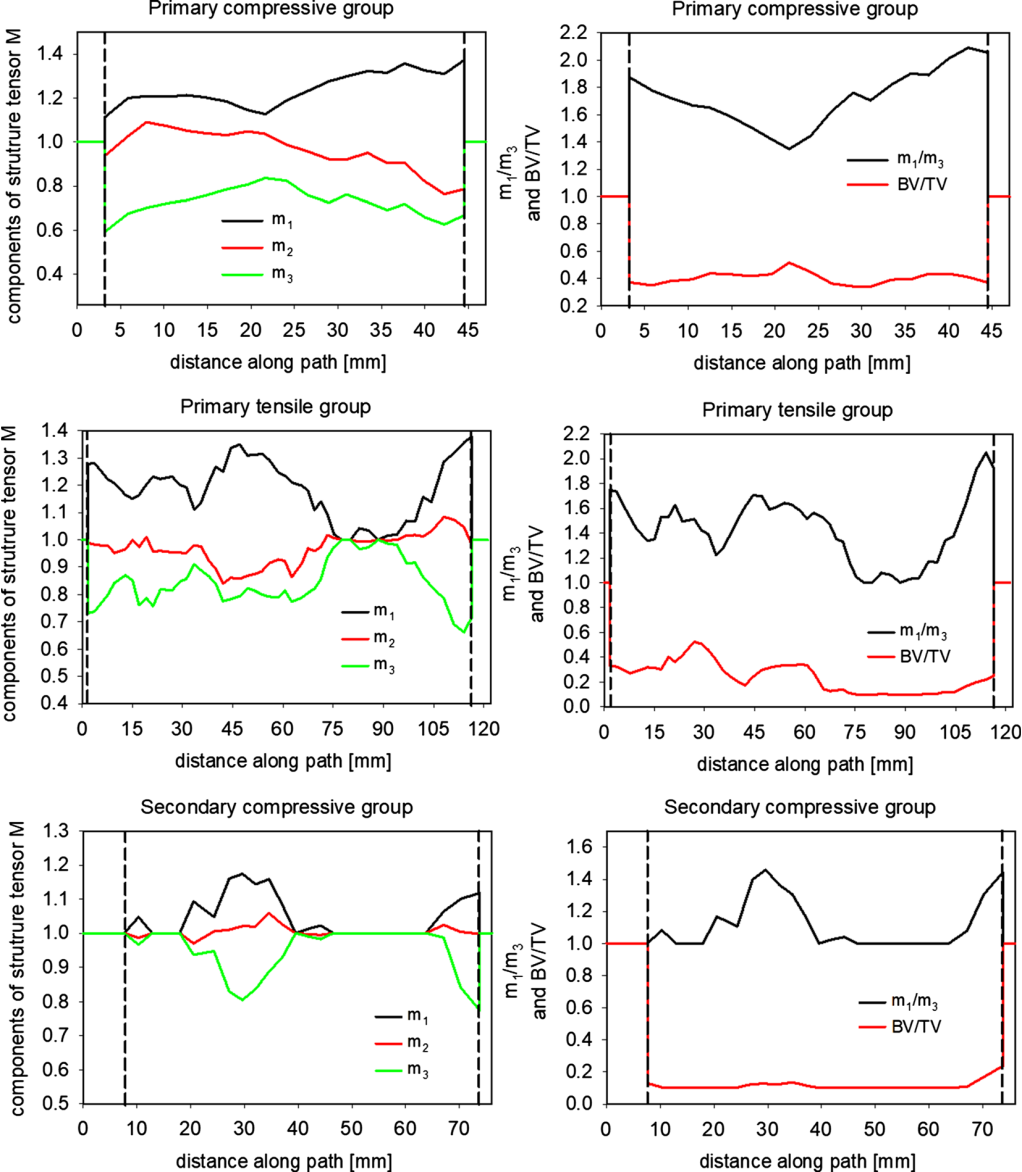
Variability of $m_{i}$ components of the structure tensor $\mathbf{M}$ and $\mathrm{BV}/\mathrm{TV}$ along various paths

This paragraph is dedicated to the analysis of the elastic anisotropy of the femoral head predicted by combining MIL method and Zysset constitutive model. Figure 11 illustrates the changes of BV/TV and eigenvalues ${m}_{i}$ of the tensor ***M*** resulting along each of the analyzed paths visualized in Fig. 5. The position on the path is specified by parameter *p*, expressed in millimeters, and corresponding to the distance from the origin of the path to a point considered on it. The beginning and end of each path involves cortical bone; therefore, at the borders the eigenvalues ${m}_{i}$ of the fabric tensor, as well as the $\mathrm{BV}/\mathrm{TV}$, are equal to 1. The vertical dotted lines in these charts delimit the thickness of the cortical tissue along each path.

When analyzing the *primary compressive group* along path (1), it can be seen (Fig. 6) that in this area, $\mathrm{BV}/\mathrm{TV}$ in the trabecular bone has a relatively high value (approximately 0.4) and that there is a considerable differentiation between the eigenvalues of the fabric tensor. This last observation indicates the relatively high anisotropy of the bone along this path.

When analyzing the differentiation in eigenvalues of the ***M*** tensor along the line running from the upper surface of the femoral head in the direction of Adams’ arch (an arch in the neck of the femur below the femoral head), it can be seen that the difference ${m}_{1}-{m}_{3}$ in the trabecular bone grows in the first 5 mm and then gradually decreases to the point of intersection with path (3) (*primary tensile group*), which is located nearby in the center of the femoral head at a distance of approximately *p* = 20–25 mm from the beginning of the first path. At this point, the density of cortical bone reaches its local maximum ($\mathrm{BV}/\mathrm{TV}\left(43\right)=0.52$). An analysis of the ratio ${m}_{1}/{m}_{3}$ is informative. Bearing in mind the dependency ${E}_{i}={E}_{o}{\rho }^{k}{\left({{m}_{i}}^{2}\right)}^{l}$ and the fact that $l$ is close to one $(l=0.99\approx 1.0)$, it is clear that the square of the ratio of eigenvalues of ***M*** is equal to the ratio of the relevant Young’s moduli:

7 $$\frac{{E}_{i}}{{E}_{j}}\approx {\left(\frac{{m}_{i}}{{m}_{j}}\right)}^{2}$$

The ratio ${m}_{1}/{m}_{3}$ attains its local minimum at the point *p* = 22 mm of greatest density (${m}_{1}/{m}_{3}\left(22\right)=1.3$, further it increases to its maximum value near Adams’ arch (*p* = 43 mm) where ${m}_{1}/{m}_{3}\left(43\right)=2.1$ and $\mathrm{BV}/\mathrm{TV}\left(43\right)=0.42$. The ratio of corresponding Young’s moduli in these points are: ${E}_{1}/{E}_{3}\left(22\right)=1.69$ and ${E}_{1}/{E}_{3}\left(43\right)=4.41$. These values indicate strong anisotropy of the bone along this path, especially around Adams’ arch. The anisotropy is caused by the very clear directionality of the trabecular bone struts which can be easily seen in Fig. 5b. Along the entire path, the component ${m}_{2}$ exhibits a slight oscillation around 1.0, resulting from the fact that direction 2 is perpendicular to the plane where the principal load occurs (it lies in the sagittal plane).

In the case of the *primary tensile group (*path 2), bone density varies from 0.105 to 0.52 and its mean value is estimated at $\mathrm{BV}/\mathrm{TV}=0.25$. The greatest density value is observed at the intersection with path (1), while the lowest value occurs between the *secondary compressive* group and the cortical bone of the lower part of the femoral head (65 < *p* < 115 mm). In the segments 78 < *p* < 98 mm of this path, all three eigenvalues *m*_*i*_ are nearly equivalent. This means that the trabecular bone is in this place quasi-isotropic and has very weak mechanical properties. The three Young’s moduli are of the order:

8 $${E}_{1}\approx {E}_{2}\approx {E}_{3}=280 \mathrm{MPA}$$

At the beginning of the path (*p* < 70 mm), the three eigenvalues of the ***M*** tensor are significantly differentiated. In turn, ${m}_{2}$ remains practically stable and close to 1 over the entire path. The ratio ${m}_{1}/{m}_{3}$ is illustrated in Fig. 11. In trabecular bone, it varies from 1 to 2.02. It achieves this relatively high value for *p* = 115 mm. At this point, the ratio of the relevant Young’s moduli is: ${E}_{1}/{E}_{3}\left(115\right)=4.08$ testifying the highly anisotropic behavior of the bone in this place. The density is relatively low $\mathrm{BV}/\mathrm{TV}\left(115\right)=0.25$ but significantly increases with growing *p*. On the initial segment of path (2), both density and the ratio ${m}_{1}/{m}_{3}$ vary considerably (0.18<$\mathrm{BV}/\mathrm{TV}<0.52$ and $1.2<{m}_{1}/{m}_{3}<1.73$). The highest value of ${m}_{1}/{m}_{3}$ occurs at a distance of *p* = 50 mm, located at the neck of the femur. This is an area where maximal principal tensile stresses occur. This place is particularly important because of fractures frequently occurring in the neck of the femur (Shivji et al. 2015). This type of fracture arises because of the hip impacts (drops).

In the case of path (3), passing through the *secondary compressive group* and its extension, which runs from the lesser trochanter in the direction of the greater trochanter, the relative density value is quasi-constant and very low (mean $\overline{\mathrm{BV}/\mathrm{TV}}=0105$). Everywhere where BV/TV is greater than 0.1, the bone has anisotropic properties and the ratio ${m}_{1}/{m}_{3}$ is even in some places greater than 1.3. In turn, everywhere where $\mathrm{BV}/\mathrm{TV}\le 0.1$, all eigenvalues of the ***M*** tensor are equal to 1. It is a result of the assumption that for areas of bone with BV/TV ≤ 0.1, the behavior of the bone is isotropic. This outcome is obviously not exact as it can be concluded from the abrupt evolution of the curves of ${m}_{i}$ and $\mathrm{BV}/\mathrm{TV}$ in Fig. 11.

Thanks to the fact that on paths (1) and (2) the $\mathrm{BV}/\mathrm{TV}$ of the bone is higher than 0.1, it was possible to accurately determine the ***M*** tensor. On the other hand, on large part of path (3) BV/TV < 0.1. This fact leads to the isotropic constitutive relation according with the Zysset-Curnier model assumption. As this study concentrates on the analysis of the impact of anisotropy of elastic properties of the bone on its internal mechanical state, in the following section of this paper only paths (1) and (2) were considered.

### Stress and strain analysis along the trabeculae groups

**Fig. 12:**
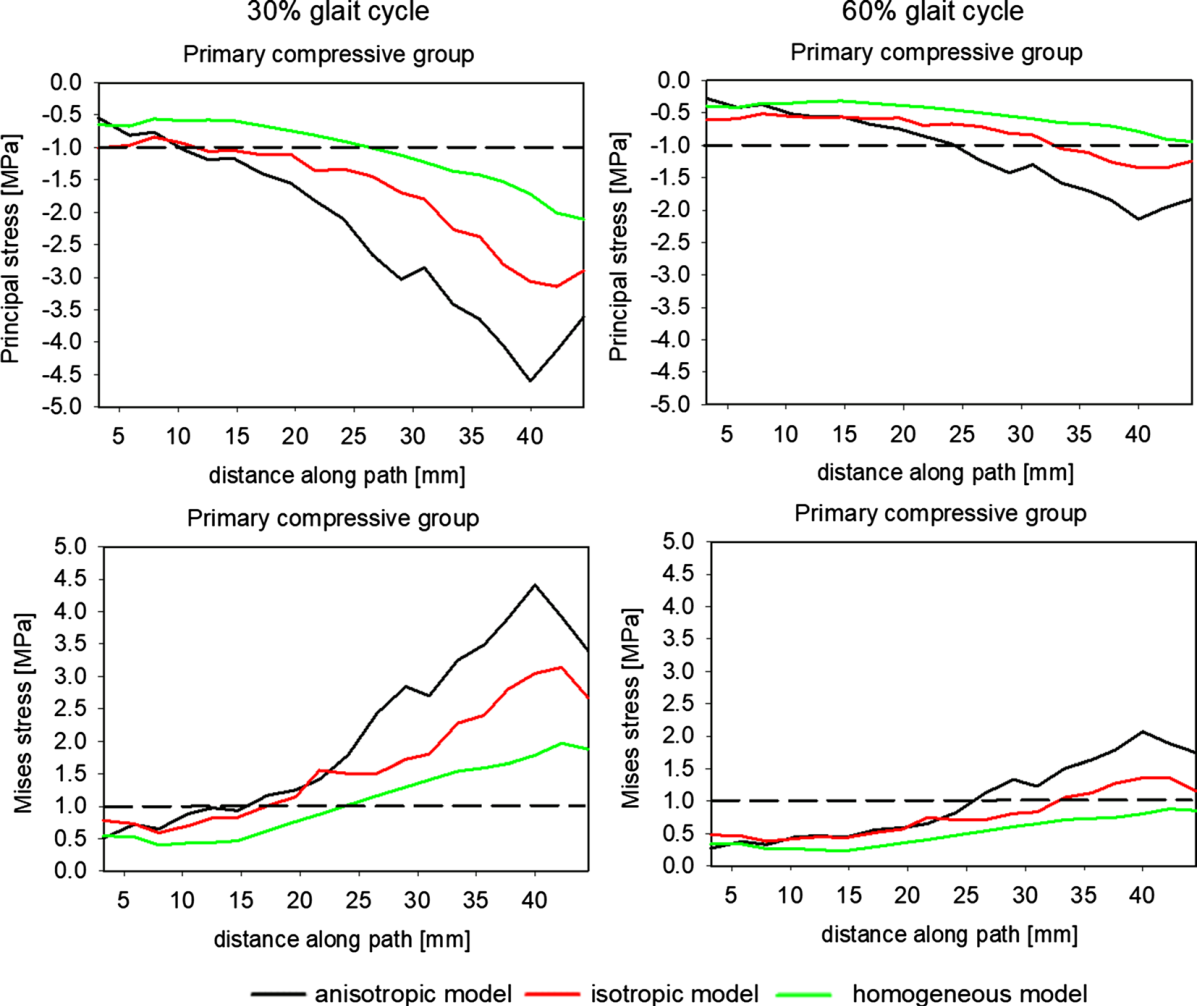
Evolution of the major principal and equivalent von Mises stress along path 1 in the primary compressive group for two leg configurations corresponding to 30% and 60% of the gait cycle

As the inherent trabecular groups occurring in the femoral head play a crucial role in transferring external load, an analysis was made of the internal state of the bone along the paths passing through these groups. The MESr threshold, introduced by Frost (Frost 2003) (see Table 4), is also indicated in this figure by dotted lines. A quantitative comparison of the state of stresses for the three models is presented in Fig. 12.

Two columns of this figure refer to two bone configurations studied. The figure shows the evolution of the major principal stress and von Mises stress along the path passing through the primary group.

The notion of equivalent stress was first introduced by Huber (1904) for isotropic materials. The equivalent stress is proportional to the part of the elastic strain energy density due to the change of shape of the elastic isotropic continuum. Thus, the physical sense of this notion is lost in the case of orthotropic (anisotropic) materials. In such a case, the equivalent stress proposed by (Lipinski et al. 2021) should be used. Their definition generalizes the definition of equivalent stress proposed by Hill (1998). Despite this remark, and because in many studies researchers utilize this stress measure for bones, Fig. 12 compares the equivalent von Mises stress along paths (1) for the three constitutive models of trabecular bone to allow for a comparison of our results with those obtained by other authors. An analysis of these results reveals, that as we move along path (1) from the femoral head to Adams’ arch, there is an increase in compressive stress for both configurations. The highest stress gradient was obtained for the anisotropic model and the lowest for the homogeneous one. The discrepancies among the models’ predictions increase when approaching Adams’ arch. In the limb configuration of 30% of the gait cycle, the difference in major principal stress, in Adams’ arch, between anisotropic and homogeneous isotropic models riches 3 MPa. The anisotropic model predicts 4.5 MPa when the homogenous model leads to 1.5 MPa at the same point *p* = 40 mm. It should also be underlined that the analyzed configuration (30% of the gait cycle) is not the leg position at which the femoral head is subject to the greatest reaction force during the gait cycle. Simulations performed with the OpenSim packet show that the greatest reaction force in the hip joint occurs when it is slightly angled backwards during the toe-off phase. Because the limb is angled backwards, the considerable stresses appear in the coronal plane but also in the sagittal one.‬‬‬‬

The correlation between the von Mises and major principal stresses (${\upsigma }_{vM}\approx -{\upsigma }_{I}$) demonstrates that the compressive stress dominates on this path. It is also worth noting that both stress measures are outside from the dead zone (1–20 MPa), for a large part of the path. This does not, however, mean that the tissue is in the disuse window introduced by Frost’s in its mechanostat theory (Frost 2003). Indeed, the quantities illustrated in this figure are overall stress measures, which obviously are lower than stresses carried by the bone tissue. Somewhat lower stresses are observed for the second leg configuration. In certain areas of the path, these stresses are less than 0.5 MPa and their maximum does not exceed 2 MPa. The lack of contact with the ground means that the loads to which the bone is subjected at this configuration are a result solely of muscular actions and the gravity force on the suspended limb.

**Fig. 13:**
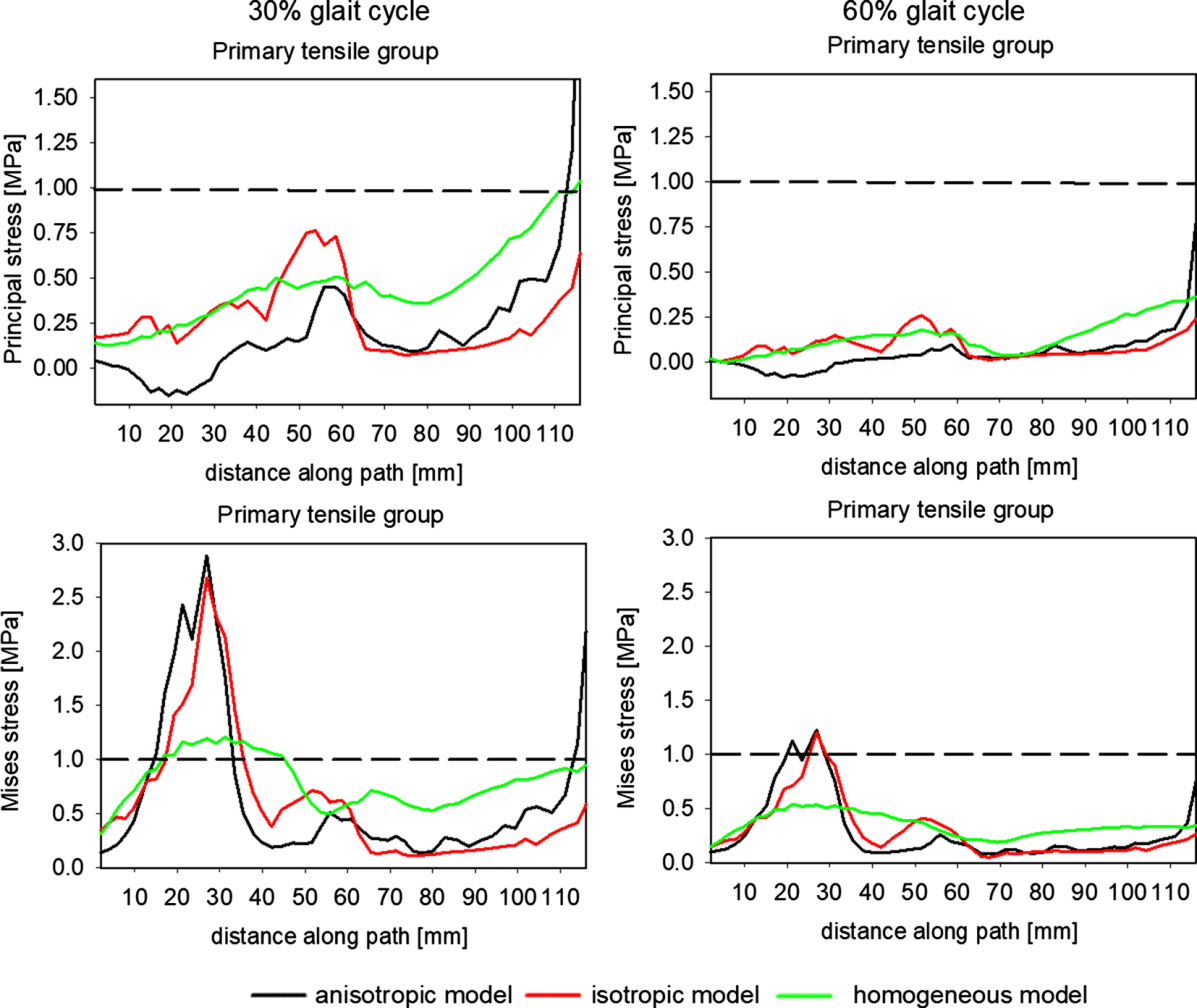
Evolution of the major principal and equivalent von Mises stress along path 2 in the primary tensile group for two leg configurations corresponding to 30% and 60% of the gait cycle

The primary tensile group is a stripe of trabeculae in which highly significant changes of BV/TV ratio and bone anisotropy are observed (see Fig. 11). This is caused by the fact that this group intersects the primary compressive group, which is characterized by high BV/TV ratio and strong anisotropy, and the secondary compressive group, which in contrast has very low BV/TV values. Figure 13 shows the evolution of major and equivalent stresses on path (2) representing this group. The form of the graphs of stresses for both limb configurations (30% and 60% of the gait cycle) are very similar, although the stresses at 60% of the gait cycle are considerably lower than those at 30% of the gait cycle. Both isotropic models (heterogeneous and homogenous) predict similar results. The major principal tensile stress predicted by these models is higher than the values obtained from the anisotropic model. A distinct maximum can be seen at around *p* = 45–60 mm. This position corresponds to a part of the femoral neck characterized by the high value of the ratio ${m}_{1}/{m}_{3}$ indicating highly anisotropic tissue. A sharp maximum is visible for the isotropic model with homogeneous BV/TV distribution. The isotropic model with a mapped BV/TV predicts “smoother” evolution of this stress. A local maximum appears also in the case of the anisotropic model, but the maximum of principal stress is nearly two times lower than in the homogeneous model. It is worth noting that the three models predict stresses lower than the MESr.

**Fig. 14:**
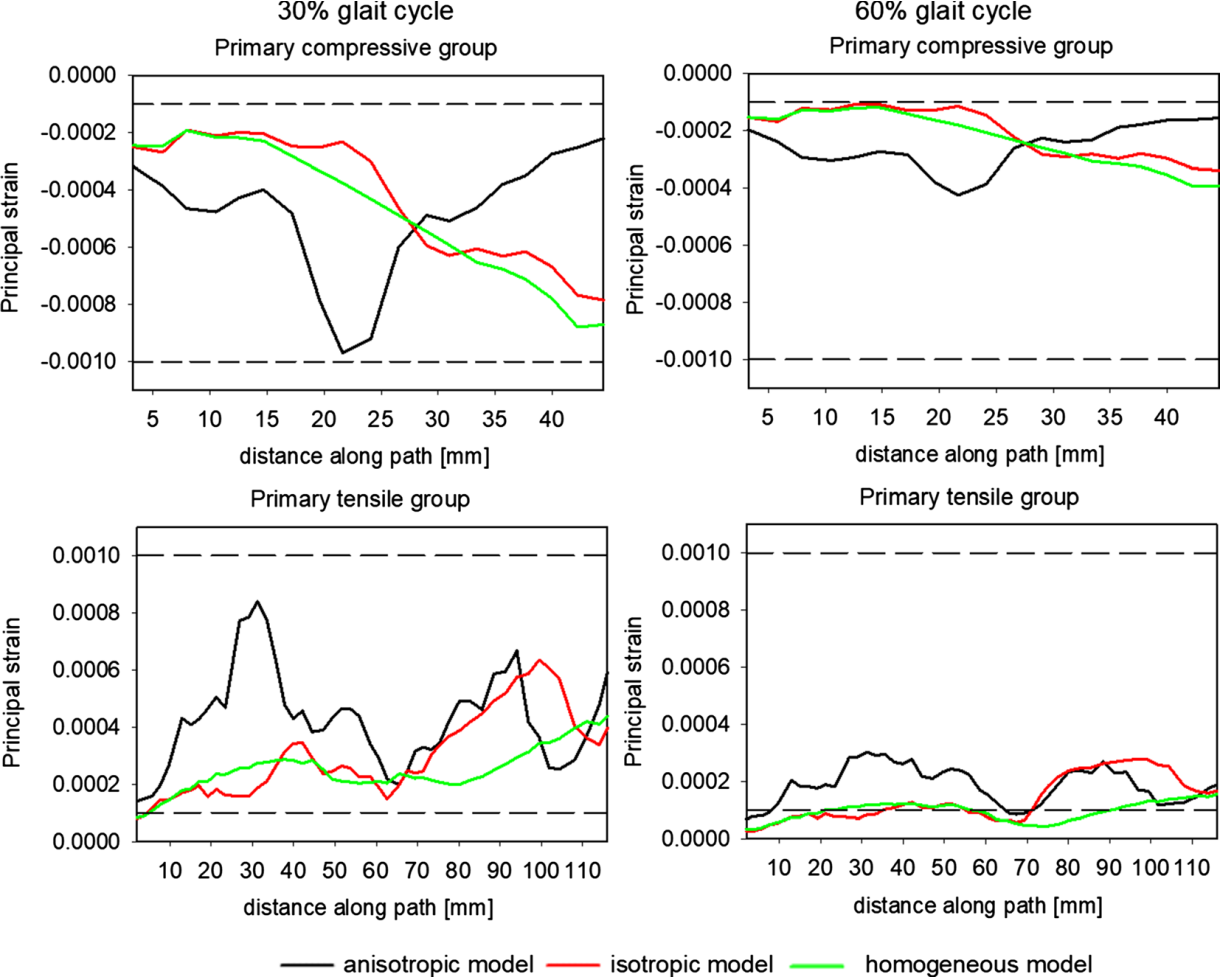
Evolution of the major principal strains along paths 1 in the primary compressive group and 2 in the primary tensile group for two limb positions corresponding to 30% and 60% of the gait cycle

A quantitative analysis of strains on primary compressive and primary tensile groups of trabeculae is shown in Fig. 14. In the primary compressive group, both at 30% and 60% of the gait cycle, the major strains predicted by the three models are mainly located within the limits of the dead zone proposed by Frost. However, it should be noted that there are large differences in the values of the strains forecast by the models. At first leg configuration, the anisotropic model predicts an increase in the absolute value of strain along the segment *p* = 15–30 mm. This increase is not anticipated by the two other models. The maximum strain here is of the order of 0.00095. Its position corresponds to the intersection of paths (1) and (2). This is the area where the greatest variation of BV/TV ratio occurs but also this is the site of high bone anisotropy (see Fig. 11.) The two isotropic models foreseen similar evolutions of major principal strain characterized by a quasi-monotonous increase in this strain along the entire path. The maximal (in absolute) values are lower than those resulting from the anisotropic model, and riches −0.0007, −0.0008 at the end of the path. Similar evolution in the distribution of major principal strain was obtained for the second limb configuration but its level is 2.5 times lower.

In the case of path (2), the major principal strains are positive. Their courses are more complex. The three models predict three different evolutions on this path (2). The anisotropic model forecast two maxima, one around *p* = 30 mm and a second one for *p* = 90 mm. The former is the place of intersection with the primary compressive group trabeculae while the latter belongs also to the secondary compressive group. This group is characterized by a very low BV/TV ratio. Once again, significant differences can be seen among the models’ predictions. The highest values of strains for these two sites are predicted by the anisotropic model. In the case of the homogeneous isotropic model, these two maxima are practically invisible. The heterogeneous isotropic model also predicts two local maxima, but they are lower in values of strain and shifted into the end of the path. It is worth noting that the anisotropic model predicts locally more than two-fold higher strains than the isotropic models. Similar observations can be made for graphs corresponding to the second limb configuration (60% of the gait cycle). The principal difference involves the level of strains occurring which is, for this configuration, nearly three times lower.

As for the first path, the major strain is mainly located in the dead zone identified by two dotted lines in this figure. According to Frost’s model, the bone should not be subject to change its density, which is in contradicts with the observations concerning principal stresses. This apparent contradiction can be easily explained by the internal structure of the bone in these trabecular groups. The trabeculae in the groups analyzed are arranged parallel to each other. This means that, to a fair degree of accuracy, the local and global (in the sense of mean field theory) strains occurring in these groups are of the same magnitude. However, local and global stresses are fundamentally different. It can be said that local stresses are significantly greater than the global ones.

### Indications concerning the choice of mechanical stimulus for bone adaptation simulations

**Fig. 15:**
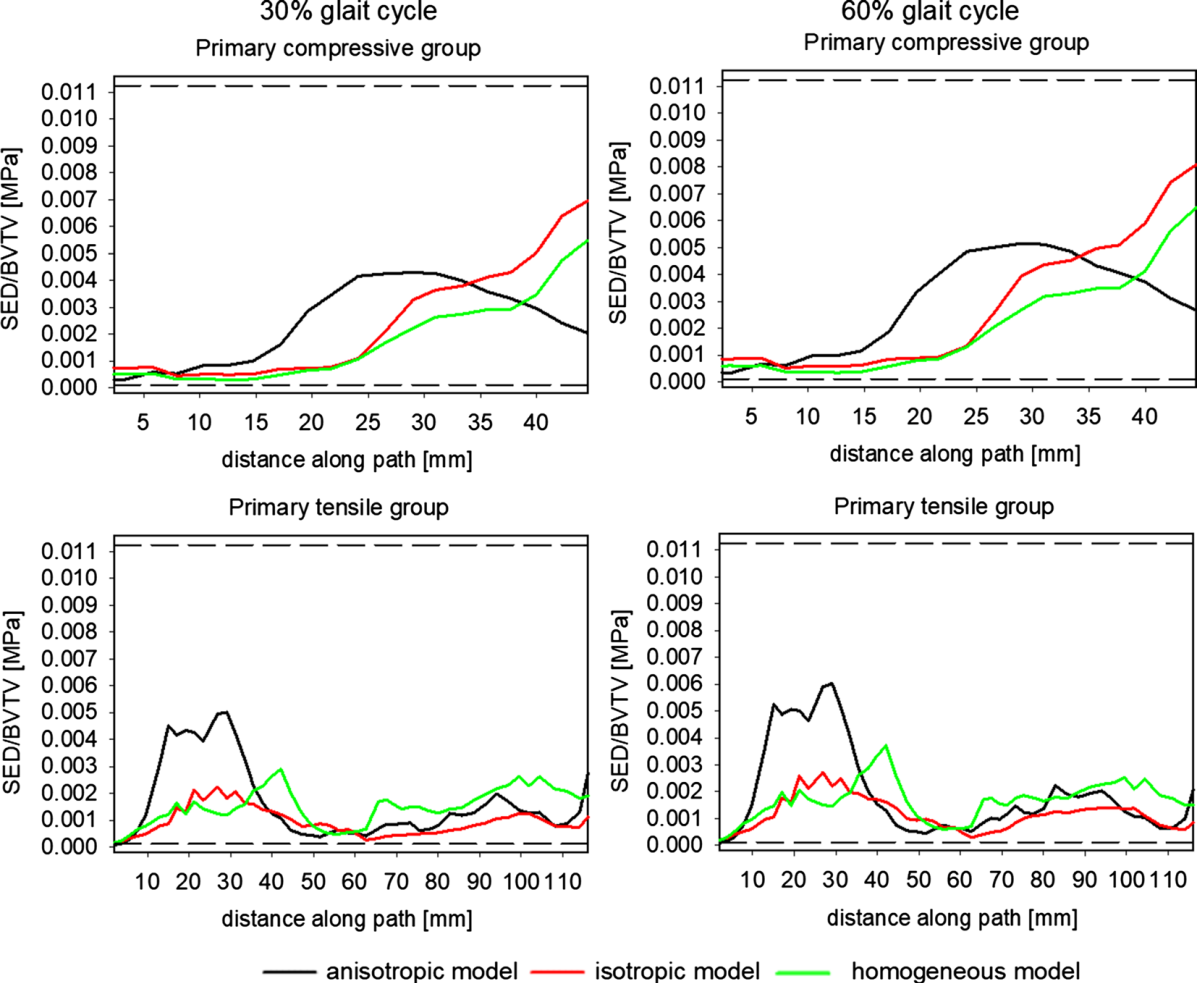
Evolution of elastic strain energy density along paths 1 and 2 for two limb positions (30% and 60% of the gait cycle)

Figure 15 presents the evolution of Strain Energy Density (SED), rescaled with BV/TV of concerned point, along paths (1) and (2) for both configurations of legs. Dotted lines in Fig. 15 indicate the thresholds of the energy density: MESr = 1.125 10^–4^ MPa and MESm = 1.125 10^–2^ MPa (see Table 3).

This figure testifies that, on two paths and for the three models and both leg configurations, the SED rests in the Frost’s dead zone. This observation suggests that the elastic strain energy density, and an appropriate measure of equivalent elastic strain associated with it, are better candidates for the mechanical stimulus than the equivalent stresses for instant such as von Mises invariant. Indeed, assuming the correctness of the Frost mechanostat theory and supposing that the analyzed bone was in a state of balance, the results obtained indicate that stresses (principal or equivalent) are not the good candidate for mechanical stimulus, see Figs. 6, 7, and 13. Namely, along a significant part of both paths, and for both limb configurations, the stress level is below the MESr threshold, contradicting the conjecture of the state of balance of the bone. This observation is particularly valid in the case of the homogeneous isotropic constitutive model. On the other hand, values of major strain and elastic strain energy density for the anisotropic model presented in Figs. 14 and 15 fall between the thresholds MESr and MESm. In the case of the homogeneous isotropic model, this observation is not entirely factual on the path belonging to the primary tensile group of trabeculae. Indeed, for the limb configuration corresponding to 60% of the gait cycle, the major strain is lower than MESr.

Presented results thus show that both measures (major strain and elastic strain energy density) are better candidates for the mechanical stimulus in the mechanostat theory than stresses are. It should be remembered, however, that the results obtained here present only one case of bone loading. Remodeling and the adaptation of the bone is the result of the action of complex dynamic (or transient) loads in which not only the value of the stimulus itself is important, but also its frequency of occurrence (Villette and Phillips 2018).

## Conclusions

This paper presents the results of static simulations of the human femur using the finite elements method subject to two loads specific for two particular positions of the right leg during the gait cycle (walking). In order to determine these physiological loads, a simulation of walking was conducted using the inverse kinematics method for the entire gait cycle. This simulation generated files of time dependent vectors of muscular forces as well as reaction forces in the hip joint constituting a reliable loading of the bone.

Two leg positions were selected for analysis for which the femoral bone appeared parallel to the axis of the body. A FE model of the femoral head subject to the appropriate muscular and reaction forces was developed from the tomographic data of the bone, three constitutive models of trabecular bone were compared; anisotropic model based on Zysset's approach, with mapped BV/TV fraction and local orthotropic bases, isotropic model with mapped BV/TV fraction and isotropic model with uniform mean BV/TV fraction and corresponding Young’s modulus. Of these three approaches, the most advanced is the heterogeneous orthotropic model, based on Cowin’s theory, in which the anisotropic properties are correlated with bone mass spatial distribution.

The simulations demonstrated that differences between the predicted results of these models are significant. Only the anisotropic model allows for the plausible distribution of stresses along the main trabecular groups. The largest differences were observed in areas of strong anisotropy of the bone which occurs in the primary compressive and primary tensile groups. In these trabecular groups, according to the Zysset model, the ratio between the greatest and smallest Young’s moduli can exceed 4. This anisotropy has a significant impact on the states of strains and stresses and, as a result, on the level of strain energy density. On path (1), which is the most loaded area of the bone, near Adams’ arch, the relative difference between the major principal stresses predicted by the anisotropic model and the homogeneous isotropic model locally exceeds 100% for both limb configurations. A similar conclusion can be reached concerning the major strain along this path. However, in this case the greatest differences occur approximately at the halfway point of the path where the trabecular primary compressive and tensile groups intersect in the femoral neck. This area is essential, as fractures of femur often occur here. According to the numerous studies presenting experimental data and numerical calculations (Mondal and Ghosh 2017; Tianye et al. 2019; Tano et al. 2019), this area often sees the initiation of fractures in a plane which, in the majority of cases, passes through the neck of the femur. The simulations presented show that the isotropic constitutive model provides underestimated values of stresses and strains for many areas of bone. Many computation packets, which are commonly applied by physicians and bio-mechanicians, whether for bone load or for the impact of endoprosthesis of the hip joint on the stress distribution, make precisely use of this approach. These include such widely used packets as Materialize Mimics https://www.materialise.com/en.), Synopsys ScanIP (https://www.synopsys.com/simpleware.html), MITK-GEM (http://araex.github.io/mitk-gem-site/) and Bonemat (http://www.bonemat.org/). All these software assume a relationship between brightness of CT (generally expressed in the Hounsfield scale) and density to determine Young’s modulus value for isotropic modeling. These packages are also frequently used in designing the geometry of implants and in preparing and predicting the effects of hip replacement operations.

Assuming the correctness of the Frost mechanostat theory and supposing that the analyzed bone was in a state of balance, the results obtained indicate that stresses (principal or equivalent) are not the good candidate for mechanical stimulus. This conclusion is particularly valid in the case of the homogeneous isotropic constitutive modeling. On the other hand, values of major principal strain and fall between the thresholds MESr and MESm for the case of anisotropic and heterogeneous model of porous bone.

Until recently, limited capacities of computers prohibited the anisotropic and heterogeneous modeling of biological organs with complex internal microstructures and subjected to realistic loads. Currently, the computational facilities allow for the use of high-definition FEM meshes and precise materials data. Despite this, much software for medical analysis still use isotropic constitutive models for predicting changes taking place in bone during modelling. The increasingly common use of high-resolution tomographs has meant that the data necessary for analysis are of considerably better quality. This fact in turn makes possible the wider use of more realistic anisotropic and heterogeneous material modelings.

## Data Availability

Not applicable.
